# A survey of rift valley fever and associated risk factors among the one-humped camel (*Camelus dromedaries*) in Sudan

**DOI:** 10.1186/s13620-016-0065-6

**Published:** 2016-06-10

**Authors:** Maiy M. M. Abdallah, Ibrahim A. Adam, Tamadur M. Abdalla, Sanaa A. Abdelaziz, Mohamed E. Ahmed, Imadeldin E. Aradaib

**Affiliations:** 1 Molecular Biology Laboratory (MBL), Department of Clinical Medicine, Faculty of Veterinary Medicine, University of Khartoum, P.O. Box 32, Khartoum North, Sudan; 2 Center for Viral Hemorrhagic Fevers, Al-Neelain Institute of Medical Research (NIMR), Al-Neelain University, Khartoum, Sudan

**Keywords:** Epidemiology, Survey, Camels, RVF, cELISA, Sudan

## Abstract

**Background:**

Rift valley fever (RVF) is a mosquito-borne viral disease of domestic livestock and wild ruminants. In camels RVF may cause abortion among pregnant camels, but is most often asymptomatic among other camels. In this study, a seroepidemiological survey was conducted to determine the prevalence of RVFV antibodies and to identify the potential risk factors associated with RVFV seropositivity among the Sudanese one-humped camel (*Camelus dromedaries*) in Khartoum State, Sudan. A cross sectional study was conducted in Khartoum State, Sudan, in a total of 240 camels selected randomly from four localities. Sera sampled were tested for the presence of RVFV-specific immunoglobulin G (IgG) antibodies using a competitive enzyme-linked immunosorbent assay (c-ELISA).

**Results:**

RVFV seropositivity was recorded in 23 out of 240 animals, prevalence rate of 9.6 % among camels in Khartoum State. Age (OR = 8.29, *p*-value = 0.04) and heavy rainfall (OR = 5.36, *p* value = 0.01) were recorded as potential risk factors for contracting RVF.

**Conclusions:**

Older age and heavy rainfall were considered as potential risk factors for seropositivity to RVF. Surveillance for RVF among camels and distribution of mosquito vectors should continue to better understand the clinical signs associated with RVFV infection in camels and provide public health authorities an opportunity to anticipate and prepare for a possible RVF outbreak in Khartoum State, Sudan.

## Background

Rift Valley fever (RVF) is a mosquito-borne viral disease that typically occurs in various areas of sub-Saharan Africa, where virus activity varies from a low-level enzootic cycle to explosive outbreaks covering large areas [1]. RVF is caused by RVF virus (RVFV), a member of the *phlebovirus* genus in the family *Bunyaviridae*. Periodically, RVFV spreads to other areas, including northward into Egypt in 1977 and eastward across the Red Sea into Saudi Arabia and Yemen in 2000 [2–6]. RVF in livestock can be devastating to agricultural communities and causes almost fatal clinical disease among young animals and high abortion rates among livestock [7]. In contrast, RVF is asymptomatic in humans and can cause a mild febrile illness. Few cases (1–2 %) progress to more severe disease, such as acute hepatitis, encephalitis, retinitis and/or a hemorrhagic syndrome [8]. The first outbreak of RVF in Sudan was reported in 1973 among sheep and cattle in the White Nile State [9]. Subsequently, RVFV was isolated from a herd of cattle in Hilat Kuku, Khartoum North [9, 10]. Serologic surveys have detected RVFV antibodies in various species of domestic livestock and in humans from different States of the Sudan, including Nile Valley, Khartoum, Kassala, El Gezira, Sennar, and White Nile [1, 11–15]. A recent seroepidemiologic survey reported a high prevalence of RVFV IgG among febrile patients admitted to New Halfa Hospital in Kassala State, Sudan [16]. New Halfa is an extensively irrigated agricultural province, located approximately 500 km east of Khartoum State. The high prevalence of RVFV- specific IgG antibodies suggested considerable circulation of RVFV in Kassala State at a particular point in time in the past.

Currently, little is known about the prevalence of RVF in livestock in Sudan and no information is available in regard to the potential risk factors associated with RVF among the livestock in the country, including the Sudanese dromedary camels. Circulation of RVFV in camels and detection of RVFV-specific antibodies is well documented in the different region of the African continent, including Sudan, Saudi Arabia, Tanzania, South Africa, Mauritania, Kenya, Niger and Nigeria [7, 17–27]. Thus, during RVFV infection, viremic camels can provide virus for vector transmission to humans and highly susceptible young ruminants, particularly lambs [24, 28]. In addition, previously employed techniques for the detection of RVFV antibodies in Sudanese camels includes agar gel immunodiffusion test, which is far less sensitive than ELISA assay and is complicated by cross reaction with other phleboviruses. Therefore, the control of RVF would be important in the Sudan given the large numbers of camels in the country, and their importance to the national economy and rural communities [1]. Most of the existing data about the epidemiology of RVF in camels in the Sudan is old. We believe epidemiologic studies, including implementation of improved surveillance, are urgently needed to better predict and respond to RVF outbreak among camels in the Sudan. The objectives of the present study were to estimate the prevalence of RVF, as determined by detection of RVF-specific IgG antibodies, and to identify the potential risk factors associated with the diseases among the one-humped camel *(Camelus dromedarius)* in Khartoum State, Sudan. This study would be expected to reduce the impact on the livelihood of pastoral communities and ultimately avoid disease spread in human population.

## Methods

### Study area

**Fig. 1:**
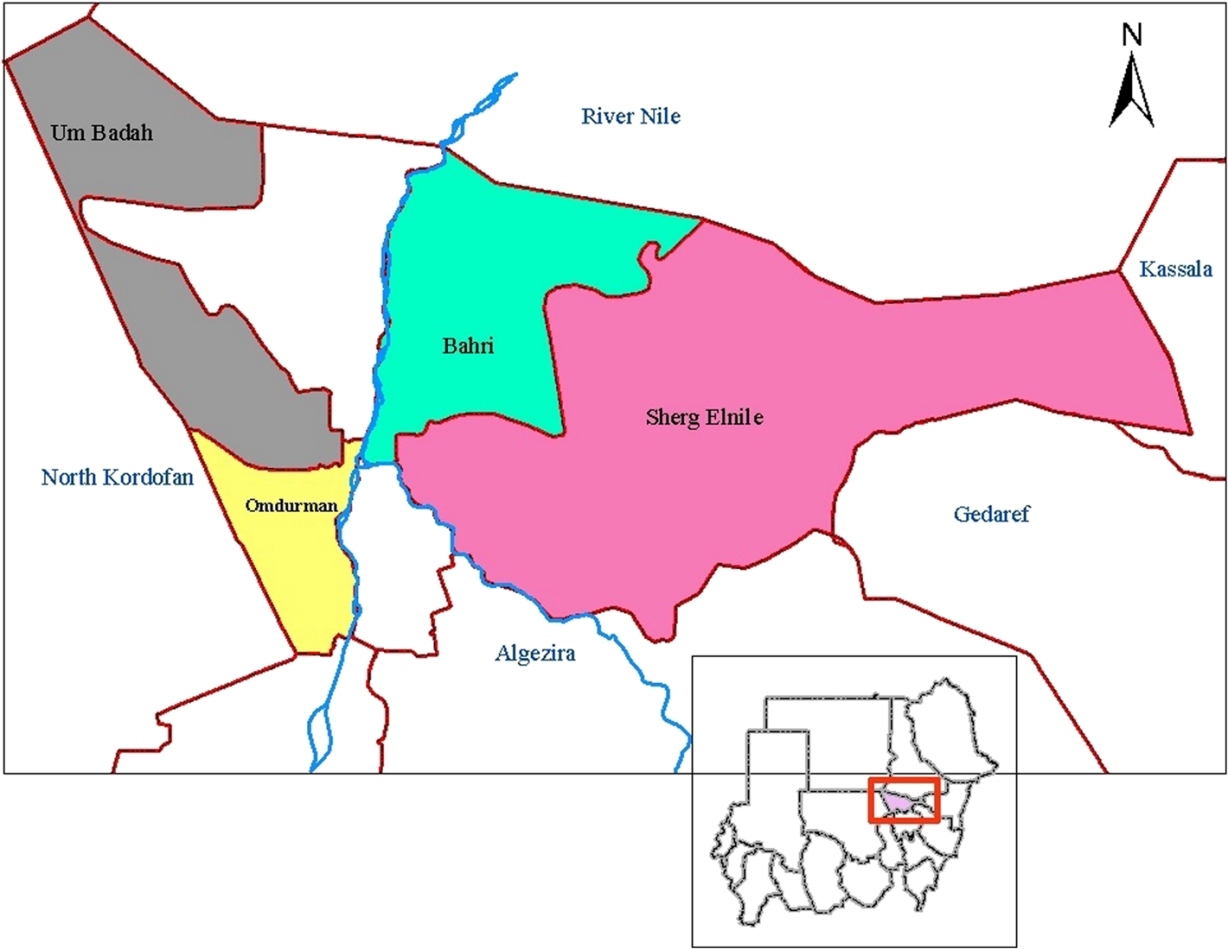
Map of State of Khartoum, Sudan showing the four localities included in the study

The study was conducted in Khartoum State during the period from October 2014 to March 2015. The State covers an area of approximately 20,971 square kilometers. The State is located between latitudes 15° 8° - 16° 39° N and longitudes 31° 36 - 34° 25 E in the semi desert tropics. It is dominated by semi desert climate, which is characterized by very hot/dry summer and cold winter. The average temperature ranges from 21 °C in the winter to 47 °C in the summer. The mean annual evaporation rate is 7.7 mm/day, and the average relative humidity ranges from 21 % to −38 %. Khartoum State is boarded by the River Nile State in the north; Kassala States in the east, North Kordufan in the west and Elgezera State to the South. The total population of camels in the country is approximately 4.6 million. The camel population of Khartoum State is 6,585 as estimated by the Sudan Ministry of Animal Resources, 2006. A map of the localities included in the study area of Khartoum State is presented in (Fig. 1).

### Study design

A cross sectional study was conducted to estimate the prevalence rate of RVFV-specific IgG antibodies in camels and to investigate the potential risk factors associated with the disease. A total of 240 camels were selected randomly using multistage probability sampling method. Four localities were randomly selected from all seven localities in Khartoum State, Sudan, which include, Um Badah, Omdurman, Sherq Elnile and Bahri. Two administration units were selected randomly from each locality. Finally, simple random sampling was applied to choose the animals from each herd [29]. All camels included in this study were aged over one year. The study is reported in compliance with the STROBE statement [30].

### Questionnaire

A pre-tested structured questionnaire with the primary objective of elucidating the multifactorial background of disease was conducted in an interactive manner at all selected herds. All animals included in this study were subjected to a questionnaire, which was filled out by the animal owners. The questionnaire include individual risk factor attributes including age (younger animals <2 years, older animals 2 years and above), sex (male, female), breed (Anafi, western), body condition (emaciate, fat), and management risk factor attributes including herd size (small, medium, large), grazing system (nomadic, semi-nomadic, stationary), rain fall (high, low), mosquito vector (presence or absence), mosquito control (practiced or not), vegetation (low, high), water bonds (presence or absence), and the four localities included in the study.

### Collection of blood samples

A total of 240 blood samples were collected from the dromedary camels in the study area of Khartoum State, Sudan. Serum samples were separated and were kept frozen at -20 °C for detection of RVFV-specific IgG antibodies using competitive enzyme-linked immunosorbent assay (cELISA).

### Competitive Enzyme-Linked Immunosorbent Assay (cELISA)

A competitive enzyme-linked immunosorbent assay (cELISA) was performed, as described by Hassanain et al. [16], using a commercially available RVFV antibody cELISA Kit (IDVet, Rue Louis Pasteur, Grabels, France). The sera were screened for RVFV-specific IgG antibodies as described by the manufacturer’s specifications. cELISA was performed in 96-well antigen-coated microplates. Unless stated otherwise, 50 microliters (μl) test volumes were used in the cELISA assay. The incubations were performed for 15 min at room temperature (23 ± 2 °C). The plates were washed three times with the provided washing buffer. Briefly, aliquots of 50 μl test sera as well as positive and negative controls sera were transferred undiluted to the RVFV antigen coated plates, using multi-channel pipette. After incubation, the plates were washed, and 50 μl of antibody-peroxidase conjugate were added to each well. The plate was incubated at 15 min at room temperature. The plates were then washed and 50 μl the substrate was added to each well. The reaction was stopped using 50 μl of the stopping solution. The results were read by using ELISA reader set at 450 nm. A presumptive diagnosis was made when the test samples produced an optical density < 50 % of the mean of the negative controls. The test samples were considered negative if the optical density ≥ 50 % of the mean of the negative controls.

### Statistical analyses

The data collected were entered into a computer on a Microsoft Excel spread sheet. Statistical analysis was performed using SPSS version 16 (for Windows) and double checked before analyses. Associations between the outcome variable (status of RVF seropositivity in camels) and its potential risk factors were first screened in a univariable analysis using Chi-square (*χ* ^2^) test. A multivariable model for the outcome variable was constructed using logistic regression analysis with enter method for modeling checking. RVF was considered as the dependent variable and the risk factors as independent variables. Finally, odd ratios and 95 % confidence interval (CI) were calculated, and risk factors with a *p-*value < 0.05 were taken as significant association to RVF seropositivity.

## Results

Using a cELISA assay, RVFV-specific IgG antibodies were detected in 23 out of 240 camels included in the study. The overall prevalence rate of RVFV antibodies among camels in Khartoum State of Sudan was estimated to be 9.6 %. The highest and lowest rates of RVFV seropositivity were recorded in Sherq Elnile and Omdurman, respectively. The results of the univariate analysis using chi-square test were presented in (Table 1). The final model of RVFV infection indicated that only two independent risk factors were statistically significant. Older cattle (>2 years of age) were eight times more likely to have been infected with RVF (OR = 8.29, CI = 1.05–9.66, *p*-value = 0.04) compared to young animals (<2 years of age). Heavy rainfall increased the risk of contracting RVF compared to low rainfall (OR = 5.36, CI = 1.46–19.66, *p* value = 0.01). Significant association between RVF and potential risk factors in the final model were shown in (Table 2). In contrast, there was no significant association between RVF seropositive camels and the animal sex, breed, grazing system, mosquito control, herd size, body condition, introduction of new animal to the herd, and localities.

**Table 1:** Univariate analysis for the association between potential risk factors and RVF seropositivity among camels in Khartoum State of Sudan using Chi-square test

Risk factors	Animals tested	Animals affected (%)	df	*χ*2	*p*-value
Locality			3	0.48	0.37
East Nile	60	8 (13.3 %)			
Bahry	60	4 (6.7 %)			
Omdurman	60	7 (11.7 %)			
Ombadda	60	4 (6.7 %)			
Age			1	0.03	0.031
Small	53	1 (1.9 %)			
Old	187	22 (11.8 %)			
Sex			1	0.70	0.48
Female	202	20 (9.9 %)			
Male	38	3 (7.9 %)			
Breed			2	0.78	0.82
Western	116	12 (10.3 %)			
Anafi	19	1 (5.3 %)			
Bushari	105	10 (9.5)			
Body condition			2	0.35	0.80
Emaciation	3	1 (33.3 %)			
Thin	82	7 (8.5 %)			
Fat	155	15 (9.7)			
Farm yard			1	0.085	0.065
In door	126	16 (12.7 %)			
Out door	114	7 (6.1 %)			
Grazing system			1	0.73	0.48
Stationary	181	18 (9.9 %)			
Nomadic	59	5 (8.5 %)			
Herd size			2	0.69	0.47
Small	28	2 (7.1 %)			
Medium	19	1 (5.3 %)			
Large	193	20 (10.4 %)			
Mosquitoes present			1	0.063	0.047
No	84	4 (4.8 %)			
Yes	156	19 (12.2 %)			
Mosquitoes control			1	0.78	0.518
No	193	19 (9.8 %)			
Yes	47	4 (8.5 %)			
Rain fall			1	0.008	0.005
Low	93	3 (3.2 %)			
High	147	20 (13.6 %)			
Vegetation			1	0.35	0.24
Low	124	14 (11.3 %)			
High	116	9 (7.8 %)			
Water bond			1	0.18	0.13
No	125	15 (12 %)			
Yes	115	8 (7 %)

**Table 2:** Multivariate analysis, using logistic regression model, for significant association (*p* > 0.05) of risk factors and RVF seropositivity among camels in Khartoum State, Sudan

Risk factors	OR	95 % C I	*P*-Value
Age			
Small	Reference	1.05–9.65.35	0.04
Old	8.29		
Rain fall			
Low	Reference	1.46–19.66	0.01
Heavy	5.36		

## Discussion

In recent years, the distribution and nature of RVF has changed substantially. RVF has become of great veterinary concern to dairy producers, wildlife managers and veterinary diagnosticians because of the frequent occurrence of sporadic cases and outbreaks among domestic and wild ruminants [1, 3, 5, 31–33]. Very little information is available about the epidemiology and disease potential of RVF in domestic livestock of the Sudan. The prominent clinical sign in RFV infected camel is mainly reflected by abortion storm [9]. Absence of clinically recognized symptoms of RVF infections with noticeable outbreaks of abortion may lead to underestimation of the importance of the disease in Sudanese camels. In contrast, clinical signs other than abortions were reported among camels in RVF outbreak in Mauritania, including hemorrhagic septicemia and severe respiratory distress [26]. This observation indicated that the pathogenicity of RVFV in the dromedary camels has not been investigated sufficiently. To advance beyond the current knowledge of the epidemiology of the disease, we conducted this study to determine the prevalence of RVF and associated risk factors among camels in Khartoum, Sudan, the second largest producing camel region in the world.

In epidemiological surveys, high prevalence rates of 45 % and 38.5 % for RVFV seropositivity were reported among camels in Tanzania and Mauritania, respectively [26, 27]. In Niger, high prevalence rate of RVFV seropositivity of 47.5 % was also reported in camels in some region of the country [34]. In Nigeria, earlier serological surveys indicated that RVF infection is generally widespread among camels with a prevalence rate of 3.3 % [19]. In the present study, the seroprevalence of RVFV-specific antibodies in camels of Khartoum State (9.6 %) is markedly higher than previously reported prevalence rates in other states of Sudan [12]. The high seroprevalence rate of RVFV in Khartoum could be attributed to newly constructed irrigation projects and agricultural scheme in the region, which might contribute in suitable climatic condition for survival of the adults and larvae of *Aedes* vectors in this region [35, 36]. In addition, the heavy rainfall associated with the large RVF outbreaks in Sudan 2007 and 2010 were suggested to contribute to high prevalence of RVF among the Sudanese dromedary camel [1, 28]. The circulation of RVFV in the country and the risks these infected camels pose for highly susceptible young ruminants necessitates the importance of improved surveillance system for this viral pathogen in Sudan. The RVF seropositivity increased with older age in the studied herds. When assessing age as a risk factor, there was a significant association between RVFV infection rate and increasing age of the animal. It was shown that the camels started to get infected with RVFV after the age of 2 years. At this age, the animals are usually released into the pasture for grazing, where they are likely to be exposed to infected mosquitoes and subsequent RFV infection. We believe that the association of RVF and age is probably attributed to frequent exposure of older camels to the mosquito vectors. Young camels (<2 years) are usually kept indoors and are well taken care of by the animal owners from contracting infectious diseases, particularly the insect and tick-borne infections. Our result is in agreement with previous epidemiological surveys, which reported higher risks of older camels for RVF infections [19, 27, 37]. It should be noted that the RVFV-specific antibodies detected among camels in Khartoum State indicated natural infection as vaccination is not practiced in the country for this animal species. In addition, all camels included in this study were aged over one year. Therefore, it is assumed that maternal antibodies were no longer persisted and that antibody indicated natural infection with RVF.

Heavy rainfall is another potential risk factor that affects RVFV seropositivity among camels in Khartom State. Our result is in agreement with that of the study conducted by other workers who reported association between RVF seropositivity and rainfall [24, 25]. The role of the international trade of livestock in dissemination of animal diseases should not be neglected. In this regard, live camels are exported to Egypt, from Sudan as a source of meat, for human consumption. Therefore, identification of the genetic lineage of RVFV strains circulating in a particular location would be important to follow the movement of the virus within and outside the African continent [1, 38–40].

In contrast, the risk assessment studies indicated that there was no significant association between RVF seropositivity and the rest of the individual or management risk factors included in the study. There was no significant difference between localities regarding RVF seropositivity in camels. In addition, there was no significant association between mosquito control and RVF seropositivity among camels suggesting failure of the control program against RVF in Khartoum State. Highest and lowest rates of RVFV infections were recorded in Sherq Elnile and Omdurman localities, respectively.

## Conclusions

The study provides evidence of circulation of RVFV among camels in Khartoum State, Sudan, as determined by the presence of RVFV-specific IgG antibodies using cELISA. It is recommended that further study should be conducted in sentinel camel herds to determine the incidence of new cases and to conduct RVF virus isolation attempts. Any recovered RVFV strains should be employed to determine the virus whole genome sequence and to identify the genetic lineage of the virus circulating among camels in Khartoum State. Surveillance for RVF in domestic livestock and studies on distribution of the mosquito vectors should continue to provide public health authorities an opportunity to anticipate and prepare for a possible RVF outbreak in Khartoum State, Sudan.
